# Major depressive disorder following terrorist attacks: A systematic review of prevalence, course and correlates

**DOI:** 10.1186/1471-244X-11-96

**Published:** 2011-06-01

**Authors:** José M Salguero, Pablo Fernández-Berrocal, Itziar Iruarrizaga, Antonio Cano-Vindel, Sandro Galea

**Affiliations:** 1 Department of Personality, Evaluation and Psychological Treatment, Psychology Faculty, University of Malaga, Campus de Teatinos s/n. Malaga, 29071 Malaga, Spain; 2 Department of Basic Psychology, University of Malaga, Malaga, Spain; 3 Department of Basic Psychology, Complutense University of Madrid, Madrid, Spain; 4 Department of Epidemiology, Columbia University, New York City, New York, USA

## Abstract

**Background:**

Terrorist attacks are traumatic events that may result in a wide range of psychological disorders for people exposed. This review aimed to systematically assess the current evidence on major depressive disorder (MDD) after terrorist attacks.

**Methods:**

A systematic review was performed. Studies included assessed the impact of human-made, intentional, terrorist attacks in direct victims and/or persons in general population and evaluated MDD based on diagnostic criteria.

**Results:**

A total of 567 reports were identified, 11 of which were eligible for this review: 6 carried out with direct victims, 4 with persons in general population, and 1 with victims and general population. The reviewed literature suggests that the risk of MDD ranges between 20 and 30% in direct victims and between 4 and 10% in the general population in the first few months after terrorist attacks. Characteristics that tend to increase risk of MDD after a terrorist attack are female gender, having experienced more stressful situations before or after the attack, peritraumatic reactions during the attack, loss of psychosocial resources, and low social support. The course of MDD after terrorist attacks is less clear due to the scarcity of longitudinal studies.

**Conclusions:**

Methodological limitations in the literature of this field are considered and potentially important areas for future research such as the assessment of the course of MDD, the study of correlates of MDD or the comorbidity between MDD and other mental health problems are discussed.

## Background

The scientific study of the psychological consequences of disasters has come a long way in the last decade [1,2]. Different reviews of the topic have shown that disasters are a relatively common event in western countries [3] capable of affecting the population in which they occur as a whole [4].

Of the different types of disasters, terrorism occupies a special place in the literature. Terrorism is defined as ''the intentional use of violence against one or more non-combatants and/or those services essential for or protective of their health, resulting in adverse health effects in those immediately affected and their community, ranging from a loss of well-being or security to injury, illness, or death'' [5]. The results of several revisions of the consequences of disasters have shown that terrorism may be associated with a greater risk of psychopathology than other disasters [6]. This characteristic, along with the increase in terrorist attacks that have struck various cities of the USA and Europe in recent years, have turned terrorism into a problem of interest, both for clinicians and for public health professionals.

A substantial body of research, much of which has been carried out after the September 11, 2001 terrorist attacks in New York and the March 11, 2004 terrorist attacks in Madrid, has documented the extent to which terrorism can affect the mental health of populations [3,6]. Of the specific psychiatric disorders studied, literature has been mainly focused on posttraumatic stress disorder (PTSD), with several reviews documenting the course and correlates of this disorder [for a review see [1,3]]. However, less is known about major depressive disorder (MDD).

The study of MDD may facilitate a more complete understanding of the psychopathological burden of trauma, which may help to design more effective population-level mental health interventions in the aftermath of terrorism [4,7-9]. Terrorist attacks can produce reactions of intense fear and horror and generate a profound sense of loss for the people involved, both of which may underlie the development of MDD [10,11]. Moreover, a positive association between the occurrence of stressful events and the probability of developing a MDD has been consistently documented in the literature [for a review, see [12,13]]. Therefore, it is plausible that MDD prevalence may increase after disasters. This, together with the high prevalence of MDD in the general population [14,15] and the substantial personal, social, and economic consequences of this disorder [16-18], suggests that MDD may be an important focus in the study of the psychological effects of terrorism.

However, with few exceptions [8], most of the data on MDD after terrorist attacks has been gathered in studies that also present data on other psychological problems (typically reporting MDD and PTSD jointly) and carried out in the context of a very specific event, at a given time and place, without comparing the results obtained with other prevalence rates. On the other hand, there is heterogeneity in the methodology used to assess MDD, with several studies using scales that assess the frequency or intensity of certain symptoms associated with MDD (and not diagnostic measures), that may hamper the correct prediction of expected rates of MDD. All this limits our ability to draw generalizable inferences about MDD after terrorist attacks and suggests that a systematic review may make an important contribution to the field [6].

We present a review of the empirical research focused on the study of MDD as a consequence of terrorism in two specific populations: direct victims (people who experienced the event in first person either because they were injured in the attack, or suffered material losses, or lost relatives or close friends [19]) and indirect victims (people in the general population). Two specific goals were established for the review: (a) to systematically review the results of studies that analyzed the prevalence and course of MDD following terrorist attacks and (b) to document the main correlates associated with this disorder. Our intention is to draw inferences that may help future research in the field and potentially guide the implementation of practical interventions when terrorist attacks do occur.

## Method

### Selection criteria

#### Type of event studied

Our review focused only on studies carried out after human-made, intentional, terrorist attacks, limiting our search to studies that were designed and conducted at a specific time and place and not including, therefore, investigations on the impact of other kinds of disasters (e.g., natural disasters) or chronic exposure to trauma (such as works carried out in times of war).

#### Type of population assessed

We focused our review on studies carried out in adult populations, including either persons in the general population or persons directly affected by a terrorist attack. We excluded work that focused on specific population subgroups such as emergency personnel, children, etc.

#### Type of assessment methodology used

Several studies in the field have adopted a dimensional approach, using scales that assess the frequency or intensity of certain symptoms associated with MDD. These studies preclude a diagnosis of MDD. Although some studies overcome this problem using different cut-off points to document MDD prevalence [20,21], this can lead to different conclusions depending on the cut-off point used [22] and to an overestimation of the presence of this disorder in the population [23]. Moreover, it is difficult compare the prevalence of MDD reported by investigations when a dimensional approach is used. Therefore, we only took into account the assessment of MDD based on diagnostic criteria, mainly based on the DSM international classification. Also, although some studies in the field use the term "incidence" rather than "prevalence", none of them were designed to ensure that persons were free from psychopathology before the occurrence of the terrorist attack. Therefore, and following other authors [1,3], we shall use the term prevalence in general throughout.

### Search strategy

**Figure 1:**
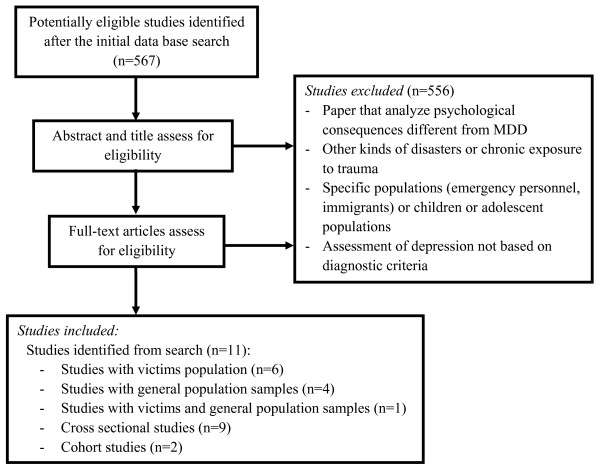
**Flowchart of the studies included in the review**.

Figure 1 presents the flow chart for the selection of the included studies. A four-step procedure was used. First, a search of the peer-reviewed literature in the PsycINFO and Medline databases was conducted (without time limit) using the following keywords: depression, terrorist, terrorism, mental health, disaster and trauma. Searches were undertaken between January 12 and 16, 2009. The initial database search identified 567 potentially eligible studies for this review. Second, two independent reviewers analyzed the title and abstracts of all retrieved studies and excluded those which did not meet the selection criteria. The majority of studies excluded in this step were papers that analyzed psychological consequences different from MDD, other kinds of disasters not categorized as terrorist attacks or other populations that were not either direct victims nor general population. Third, full manuscripts were obtained for all publications included after the second step. We examined the complete text of the articles and once again eliminated those which did not comply with the selection criteria. The majority of studies excluded in this step were papers that analyzed the psychological consequences of terrorist attacks without assessing MDD with diagnostic criteria. Fourth, to verify that our final sample was comprehensive and that our search was appropriate, we compared it with previous review papers [1,3,6].

### Search Results

Our search identified 11 studies of MDD following terrorist attacks: 6 were carried out with victims, 4 with general population samples, and one with victims and general population. Of these, 9 were cross-sectional studies and 2 were cohort studies. The most relevant information of each reviewed study is summarized in Tables 1 (studies with victims) and 2 (general population studies). In these, information about the terrorist attacks, assessment time, sample size, method and main results (MDD prevalence) is shown.

**Table 1:** Studies of major depression prevalence in victims of terrorist attacks

Study	Assessment time	Sample	Method	Instrument	Measurement	Results
Abenhaim et al. (1992)	Between 4 months and 3 years after the attacks occurred in France between 1982 and 1987	254 victims	Self-report	15 items created ad hoc for this study and based on the DSM-III criteria Questions addressed complaints such as feeling depressed, irritability, sadness, sexual difficulties, loss of appetite, or asthenia.	Current depression (past month)	13.3% [10% men; 17.7% women] * 21.8% among the severely injured 8.5% among the mildly injured or uninjured
North et al. (1999)	6 months after the 1995 Oklahoma City bombing	182 victims	Personal and telephone interview	Diagnostic Interview Schedule (DIS)/Disaster Supplement based on the DSM-III-R criteria[27]	__	22.5% [13% men; 32% women] **
North (2005)	6 months after the 1995 Oklahoma City bombing Between 8 and 10 months after the attack in Nairobi, Kenya, 1998	182 victims from the Oklahoma City bombing 227 victims from the Nairobi attack	Personal and telephone interview in the Oklahoma City study Personal interview in the Nairobi study	Diagnostic Interview Schedule (DIS) based on the DSM-IV criteria, with adjustments for cultural fit [28]	__	Oklahoma: 20.9% [11.4% men; 29.8% women] ** Nairobi: 19.4% [15.8% men; 23.6% women] *
Iruarrizaga et al. (2004)	1 month after the M-11 terrorist attacks in Madrid	117 direct victims	Telephone interview	SCID's major depressive disorder (MDD) interview [30] Diagnostic interview based on the DSM-IV-TR criteria	Current depression	31.3% [19.1% men; 40% women] **
Gabriel et al. (2007)	5-12 weeks after the M-11 attacks	127 victims who requested medical assistance	Personal interview	Mini international neuropsychiatric interview (MINI), Spanish version [29] Diagnostic interview based on the DSM-IV	Current depression (last 15 days)	31.5% ^†^
North (2001)	Follow-up 11 months after the study of North et al., 1999.	182 victims from the first assessment, 141 in the second	__	Diagnostic Interview Schedule (DIS)/Disaster Supplement based on the DSM-III-R criteria [27]	__	50% reduction in the prevalence of depression between 6 months and 1 year later ^†^
Conejo-Galindo et al. (2008)	1, 6 and 12 months after the M-11 terrorist attacks in Madrid	56 victims who requested medical assistance 44 second assessment 42 third assessment	Personal interview carried out by psychiatrist	Mini international neuropsychiatric interview (MINI), Spanish version [29] Diagnostic interview based on the DSM-IV criteria	__	One month later: 28.6% ^†^ 6 months later: 22.7% ^†^ 12 months later: 28.6% ^†^

Note: "Current depression" refers to people who suffer from major depression at the time of the interview

* Difference is not statistically significant

** Statistically significant difference

^† ^Separate rates of depression in men and women not documented

**Table 2:** Studies of major depression prevalence in general population

Study	Assessment time	Sample	Method	Instrument	Measurement	Prevalence
Galea et al. (2002)	5-8 weeks after S-11	Representative sample of Manhattan south of 110th street N = 998 adults	Telephone interview	SCID's major depressive disorder (MDD) interview [30] Diagnostic interview based on the DSM-IV-TR criteria	Current depression (last 30 days)	9.7% [7.3% men; 12% women] **
Person et al. (2006)	6 months after S-11	Representative sample of the metropolitan area of New York N = 2700	Telephone interview	SCID's major depressive disorder (MDD) interview [30] Diagnostic interview based on the DSM-IV-TR criteria	Depression since terrorist attacks Current depression (last 30 days)	Since terrorist attacks: 9.4% [7.9% men; 10.7% women] * Current: 3.9% [3.6% men; 4.2% women] *
Nandi et al. (2005)	4 months after S-11	Representative sample of New York N = 2001	Telephone interview	SCID's major depressive disorder (MDD) interview [30] Diagnostic interview based on the DSM-IV-TR criteria	Depression since terrorist attacks	9% ^†^
Gabriel et al. (2007)	5-12 weeks after the M-11 attacks	Sample of residents of Alcalá de Henares (Madrid) N = 485	Personal interview	Mini international neuropsychiatric interview (MINI), Spanish version [29] Diagnostic interview based on the DSM-IV criteria	Current depression (last 15 days)	8.5%^†^
Miguel-Tobal et al. (2006)	1 month after the M-11 terrorist attacks in Madrid	Representative sample of Madrid N = 1589	Telephone interview	SCID's major depressive disorder (MDD) interview [30] Diagnostic interview based on the DSM-IV-TR criteria	Current depression (past month)	8% [5.1% men; 10.6% women] **

Note: "Current depression" refers to people who suffer from major depression at the time of the interview; "Depression since terrorist attacks" refers to those who have suffered major depression at any given time since terrorist attacks.

* Difference is not statistically significant

** Statistically significant difference

^† ^Separate rates of depression in men and women not documented

In our review, most studies examine the impact of terrorist attacks in Madrid (March 11, 2004) and New York (September 11, 2001). Nevertheless, one study assesses the impact of different terrorist attacks occurred in France (between 1982 and 1987), another one assesses the consequences of the Oklahoma City Bombing (1995), and yet another one compares the consequences of the Oklahoma City Bombing with the attack on the US embassy in Nairobi, Kenya (1998).

The measures used to establish MDD prevalence were the Diagnostic Interview Schedule (DIS)/Disaster Supplement, based on the DSM-III-R criteria [24] (used in 2 studies); the Diagnostic Interview Schedule (DIS) based on the DSM-IV criteria, with adjustments for cultural fit [25] (used in 1 study); The Mini International Neuropsychiatric Interview (MINI), based on the DSM-IV criteria [26] (used in 2 studies); and the SCID's major depressive disorder (MDD) interview [27], based on the DSM-IV-TR criteria (used in 5 studies). In one study [28] the researches assessed MDD with 15 items created ad hoc and based on the DSM-III criteria.

## Results

### Prevalence and course of MDD after terrorist attacks

#### Results in direct victims

One of the first studies with direct victims was carried out by Abenhaim, Dab, and Salmi [28]. These authors studied the consequences of 21 terrorist attacks that occurred in France between 1982 and 1987. Data were collected between 4 months and 3 years after the attacks. Results showed an overall prevalence of MDD of 13.3%, although this depended on the degree of the effect or harm suffered by the person: 21.8% among the severely injured and 8.5% among the mildly injured or uninjured.

After the Oklahoma City bombing in 1995, which caused the death of 167 people and left more than 600 wounded, several studies were carried out with persons selected from the record of victims of the Health Department of Oklahoma. In the first study, using a sample of 182 victims, North et al. [29] found that 22.5% of them suffered MDD between 4 and 8 months after the attacks. Moreover, 56% of these reported that they had not suffered this disorder previously. In another investigation, North et al. [30] examined the prevalence of different mental disorders, among them MDD, in victims of two different terrorist attacks, the Oklahoma City bombing and the attack on the US embassy in Nairobi, Kenya, in 1998. The goal was to compare the mental health of populations exposed to terrorism in different continents--North America and Africa--using a similar methodology in both studies. Results showed no significant differences in the prevalence of MDD in these populations in both men and women. There were no differences in the prevalence of other pathologies such as PTSD or panic disorder suggesting comparable consequences of terrorist events across very different contexts.

In Spain, several studies analyzed the psychopathological consequences of the terrorist attacks of March 11, 2004, in Madrid. In these attacks, ten bombs placed on four suburban trains caused the death of 191 people and wounded approximately 1800. Between one and three months after these events, Iruarrizaga, Miguel-Tobal, Cano-Vindel and González-Ordi [31] surveyed a sample of victims who were either in the trains or at the stations where the bombs exploded, or had lost relatives or close friends, or whose relatives or close friends had been wounded. This study documented a prevalence of MDD of 31.3%. Similar results were found in two other studies that assessed a sample of victims who requested medical assistance in various Madrid hospitals on the day of the terrorist attacks, despite differences in the assessment instruments and the methodology between them. The prevalence of MDD was 31.5% in the first study [32] and 28.6% in the second [33].

Results are contradictory with regard to the course of MDD. In a follow-up study carried out by North [34] after the Oklahoma City bombing, only 50% of those who suffered MDD six months after the attack were still depressed one year later. On the other hand, after the March 11, 2004 attacks, whereas Conejo-Galindo et al. [33] found that the prevalence of MDD decreased slightly at 6 months (22.7%) they also found that the prevalence 12 months after the attacks was comparable to what it had been 1 month after the attacks (28.6%).

#### Results in the general population

Terrorist attacks can have an effect on the population that is directly assaulted or even on an entire nation [20,21]. Several studies have documented the consequences of terrorist attacks on the population as a whole.

Galea et al. [35] assessed a sample of residents of Manhattan between 5 and 8 weeks after the September 11, 2001 World Trade Center attacks. They found a prevalence of current MDD of 9.7%. These findings were replicated in another cross-sectional sample studied 4 months after the attacks [36].

Person et al. [8] assessed the prevalence of MDD six months after September 11 terrorist attacks in a representative sample of the metropolitan area of New York. Data showed that the prevalence of MDD was 3.9%, suggesting a return to baseline in MDD in the general population 6 months after the attacks.

Miguel-Tobal et al. [37] carried out an epidemiological study to document the psychological consequences of the March 11, 2004 terrorist attacks in Madrid. Using a methodology similar to the one employed by Galea et al. [35], and adapting the instruments they used, they assessed a representative sample of the adult population of Madrid between 5 and 15 weeks after the attacks. The results showed a prevalence of MDD of 8%. After the same event, similar results were found [32] in a sample of residents from the population of Alcalá de Henares (Madrid). The prevalence of current MDD in this case was 8.5%.

### Correlates of MDD after terrorist attacks

The correlates of MDD reported in the reviewed studies were classified as pretraumatic, peri-traumatic, posttraumatic, and sociodemographic factors.

#### Pretraumatic factors

Several of the studies discussed up to this point have shown that the probability of suffering MDD after a terrorist attack was increased by at least twofold among those who had experienced at least one stressful situation in the 12 months prior to the terrorist attack [8,32,35,37].

#### Peri-traumatic factors

Variables that have an impact during or some time immediately after the attack are included in this category. Among them, the emotional reaction in the immediate aftermath of the attack has been shown to be a significant predictor of subsequent MDD. Across studies, the risk of developing MDD one month after the terrorist attacks, or of still suffering from MDD six months after the terrorist attacks, is approximately three times higher in those with symptoms of panic during or shortly after the attacks [8,35,37]. Similar results were shown in the people who admitted having been afraid to die or of being injured during the attack [37].

#### Posttraumatic factors

The factors or events that occurred in the weeks or months after the terrorist attack were classified in this category. Among them, the occurrence of stressful events or the loss of psychosocial resources after the terrorist attacks is noteworthy. Having experienced more stressful situations after September 11, 2001, multiplied the probability of suffering from MDD by between 1.2 and 2.4 in a representative sample of residents of New York City [8]. In addition, the loss of psychosocial resources has been associated with MDD in other study [35].

#### Sociodemographic factors

Of all the sociodemographic variables studied, the clearest relation was found between gender and the risk of MDD following the terrorist attacks, with women having consistently higher prevalence of MDD after these events. This result has been documented in direct victims of terrorist attacks [32,33] and in the general population [32,35,37].

Other variables commonly analyzed, such as age, race, or ethnicity, do not show a consistent relation with MDD in these studies. For example, being Hispanic was a significant predictor of MDD one month [35] but not 6 months [8] after the September 11, 2001 attacks or, with respect to age, prevalence of MDD was lower in older people after September 11, 2001 attacks [35] but not after the March 11, 2004 terrorist attacks [37]. Nonetheless, variables such as the economical or educational level were not associated with a differential risk for the onset of MDD.

Several studies have assessed the proximity of residence to the place where the terrorist attacks occurred and the relation of this variable with subsequent MDD. In contrast with the findings in the assessment of PTSD [3], the proximity of one's residence has not been consistently shown to be a predictor variable of MDD, at least in the works with general population [37].

Results are inconsistent with respect to social support. Whereas in some studies the perception of social support in the months prior to the terrorist attack was shown to be a negative predictor of MDD [35,37], in other works no significant association between these variables was found [8,32].

### Overview of the excluded studies

Excluded studies that assess the prevalence of other psychological problems after terrorist attacks have generally been focused on PTSD. The research literature suggests that the burden of PTSD in persons exposed to disasters is significant. Specifically, the prevalence of PTSD among direct victims ranges between 30% and 40%, while the range in the general population is between 5% and 10% [see 3 for a review]. Furthermore, a common result is that the prevalence of PSTD in the aftermath of a natural disaster is often lower than the rates documented after human-made disasters (such as terrorist attacks) [1,3,6].

Other studies not included in our review examined the prevalence of MDD after natural disasters or chronic exposure to trauma. Some of the natural disasters evaluated have been the 1999 Turkey earthquakes [38], the 2004 Asian Tsunami [39], the 2004 hurricane in Florida [40] or the 2005 hurricane Katrina [41]. Natural disasters affect broad geographic areas, leading investigators to study populations that often include both direct and indirect victims [3]. Consequently, reports of MDD prevalence rates after natural disasters vary widely. For instance, a study on the Turkey earthquakes showed a higher prevalence of MDD closer to the epicentre (16%) compared to 100 km away (8%) [38]. Another study on the Asian tsunami found a higher prevalence of MDD in displaced people (30%) than in two other samples of non-displaced people (21% and 10%, respectively) [39]. Focused on chronic exposure to trauma, different studies have examined the significant impact of terrorism in the Israeli population since the beginning of the Al Aqsa intifada in September 2000. In a study conducted in April-May 2002, Bleich et al. [42] showed that over half of a national representative sample of Israel reported feeling depressed. In another population-based study carried out between January 2002 and December 2005 [43], 15.4% of participants reported a MDD, with rates of MDD being 2.4 times higher among Arab Israelis than among Jews. This difference between Arabs and Jews has been shown in other studies, and is consistent with both MDD as well as other psychological problems [44,45], suggesting that the mental health impact of terrorism differs among diverse groups living in Israel. Along the same line, different authors have documented the psychological impact of impending forced settler disengagement in Gaza. Hall et al. [46] assessed a sample of Israeli settlers who, after having been exposed to ongoing terrorism, were forced to leave their homes. The prevalence of MDD was 16.8%, 5 times greater than that of settlers living in the occupied territories before the Gaza disengagement. Other papers have assessed the predictors of depressive symptoms in population-based cohort studies [11,47].

Together with victims and general population, specific population sub-groups (e.g., emergency personnel or children and adolescents) have also been evaluated. In contrast to the findings in the assessment of PTSD, where the prevalence of PTSD among rescue workers is higher than in the general population [4], prevalence of MDD in rescue workers seems to be lower than in victims or in general populations, as different studies carried out after March-11 terrorist attacks have clearly shown [48]. On the other hand, the impact of terrorism in children and adolescents reveals that a substantial proportion of youth reports a wide array of clinical needs and functional impairments months after an attack [see 49 for a review]. The role of protective factors for depression in adolescents has also received attention in the literature. For example, two prospective studies documenting changes in depressive symptoms (as measured by CES-D) in Israeli adolescents exposed to missile attacks [50] and suicide bombings [51] showed that social support (mainly friendly social support) buffers the effect of terrorism-related perceived stress in predicting changes in depression.

## Discussion

The reviewed literature suggests that terrorist attacks are a risk factor for the development of MDD, mainly in the first months after its occurrence, and in certain at-risk populations. The risk of MDD ranges between 20 and 30% in direct victims of terrorist attacks and between 4 and 10% in the general population in the first few months after terrorist attacks. These prevalence rates are 2-3 times higher than might be expected according to general population surveys [14,15]. These results are consistent across studies that have used separate methodologies and assessment instruments after different terrorist attacks occurring in various cities [as in the case of the studies 35 and 37, or the study 30]. This suggests that the consequences of terrorist attacks may be universal and, in some respects at least, independent of context.

It is not easy to perform longitudinal investigations after the occurrence of terrorist attacks and the scarcity of studies in this area limits clear inference. Thus, whereas in some studies the prevalence of MDD in victims has decreased over time [34], in other studies, it has remained relatively stable [33].

There are several risk factors that have consistently been shown to be associated with the risk of suffering from MDD after a terrorist attack. These include having undergone stressful situations before or after the attack, having suffered a panic attack during the attack, being female, and having borne a greater loss of psychosocial resources. Although high perceived social support has been shown to be a protector factor for the onset of other psychological problems in several studies [1], this result is inconsistent in relation to MDD. This inconsistency in the published studies may suggest the existence of moderating and/or mediating variables in the relation between social support and MDD after terrorist attacks.

Previous literature has noted that severe levels of impairment are most likely to occur in people exposed to terrorism than to any other types of disaster, such as natural disasters [1,6]. Consistent with these observations, the prevalence of MDD reported in our review appears to be higher than that reported after natural disasters [38-41]. Terrorism has been distinguished from natural disasters by its capacity to produce greater sense of fear, loss of confidence in institutions, unpredictability and pervasive experience of loss of safety [4]. These characteristics may be associated with the increased risk of psychiatric morbidity after terrorism. However, the different rates of MDD after natural disasters and terrorist attacks may be due to differences in the samples assessed among studies. After natural disasters, it is difficult to classify persons as either direct or indirect victims [3] and, consequently, the study sample may include persons who were more or less directly exposed to the disaster [6].

Together with MDD, some of the reviewed studies assessed the prevalence of PTSD and some examined the comorbidity between MDD and PTSD. Given the evidence indicating the high rates of comorbidity between MDD and PTSD following trauma [52,53], it is likely that MDD seldom happens in isolation after terrorist attacks. In this respect, one study examined in this review [37] reported high rates of comorbidity in general population, with around 50% of individuals with MDD having comorbid PTSD one month after S-11, and around 30% with MDD having comorbid PTSD one month after M-11. Similar results were reported in direct victims exposed to the Oklahoma City Bombing (55% of subjects with PTSD were also diagnosed as having MDD) [29]. The mechanisms linking PTSD and MDD remain unclear, with alternative explanations including PTSD and MDD as a single general traumatic stress construct [54], comorbid MDD developing as a secondary reaction [55] or MDD and PTSD as relatively independent posttraumatic disorders [56]. Reviewed studies show that MDD is not always concurrent with PTSD and suggest that, consistent with previous studies carried out after other traumatic events [52,53], both disorders can be considered related but different posttraumatic reactions. In this regard, Rubacka et al. [57], examining the specific association of PTSD cluster symptoms (re-experiencing, avoidance, and hyperarousal) and MDD in a sample of mothers directly exposed to the WTC attacks, showed that only higher arousal symptom scores were significantly correlated with persistent MDD. Furthermore, if we compare the rates of MDD and PTSD found in some reviewed reports, we can reach some interesting conclusions. Whereas in direct victims the probability of developing PTSD after terrorism is higher than that of MDD (with percentages of PTSD usually over 30%), this tendency is reversed in the general population. For example, the prevalence of PTSD and MDD in the general population was 7.5% and 9%, respectively, after S-11 [35,36], and 2.3% and 8%, respectively, after M-11 [37]. These results support those found in previous research [53,56], suggesting that the pathways to MDD and PTSD may by somewhat distinct; whereas the intensity of the attack and the degree of exposure may be more closely involved in the development of PTSD, bereavement and psychosocial loss may underlie MDD after a terrorist attack [10,37].

The aim of the current work was to review the evidence regarding MDD following terrorism. There are some limitations to the literature in the field and to our review that need to be taken into account when interpreting the results herewith presented.

### Limitations of the literature in the field

First, although we only included studies that assessed MDD based on diagnostic criteria, most of them used instruments that did not include an assessment of either manic or psychotic symptoms, therefore we could not classify the disorder beyond probable MDD [8]. Although the prevalence of bipolar affective disorder is not much higher than 1% [14] it could be inflating the MDD percentages. Future investigations should take this into account in order to help improve our understanding of the psychopathological processes involved. Second, as we mentioned in the selection criteria section, the reviewed studies were not designed to ensure that persons were free from psychopathology before the occurrence of the terrorist attacks, which means that prevalence, instead of incidence, was assessed. Moreover, none of them included a control group that would enable the comparison between exposed and non-exposed populations. We overcame this challenge by comparing the prevalence of MDD following terrorism with the prevalence reported in other general population surveys. However, it is important to be cautious when interpreting this comparison because the majority of these epidemiologic studies are referred to a whole nation's population (e.g. Spain or United States) [15,58] and not to the city where terrorist attacks occurred (e.g. Madrid or New York), hence there could be differences between the two. Future studies should attempt to analyze the incidence of MDD by establishing baseline psychopathological assessments that may be used as population cohorts to document MDD incidence in the event of terrorism exposure. Third, there are several challenges facing longitudinal studies that aim to document the course of MDD. Several studies suffer from attrition, that is, the reduction in the number of people who participated in the follow-up studies. This may have biased the prevalence estimates of MDD, especially in the case of small samples [33]. Fourth, we have to be careful when extracting conclusions with respect to some correlates of MDD, mainly the pre-traumatic factors. In the reviewed studies, the assessments documented always took place after the terrorist attack in question. It is possible that pre-event reports are biased in the sense that depressed persons may selectively recall stressful situations that occurred before the attack to a greater extent than non-depressed persons.

### Limitations of the review

In relation to the characteristics of our review, we only considered studies that had assessed samples of direct or indirect adult victims. Even though we did not include studies that assessed children or adolescents, further work with this age group is clearly warranted. We also limited our search to studies that analyzed the consequences of terrorist attacks and not other kinds of disasters; knowing and comparing the prevalence and course of MDD after natural, technological, or other disasters linked to interpersonal violence (such chronic exposure to trauma) could help us understand the onset of mental disorders after mass traumatic events. Finally, we have focused our review on the examination of MDD using diagnostic criteria. This enables us to compare prevalence rates of MDD with previous epidemiological surveys and between studies carried out after different terrorist attacks. However, MDD is not the sole disorder within the unipolar spectrum and extant research after terrorism has also highlighted the high prevalence and impairment associated with other forms of depression, such as mild or minor depression. Including these other forms of depression, together with the risk factors associated with it, could be of research and public health interest.

### Implications for future research in this field

Our review highlights some key areas that are important for future research and may serve to guide intervention. First, the course of MDD after terrorist attacks remains unclear. That is why greater efforts are needed to elucidate the course of MDD after terrorist attacks. Second, there is very limited literature about psychological constructs that may be associated with MDD after terrorist attacks [3,9]. It would be interesting, in this context, to analyze the role played by other variables that have been shown to be related to MDD, such as attributional style [59], self-esteem [60] or response styles to depression [61], and to examine the way in which certain psychological variables interact with other sociodemographic variables to predict the onset of MDD. For example, it is possible to analyze which psychological factors mediate the relationship between MDD and gender. This line of research will be useful in helping to identify the persons with higher probability of developing MDD following a terrorist attack and to improve the efficacy of the interventions from which they will benefit. Third, more research is needed on the role of MDD in psychiatric comorbidity after terrorist attacks. Although some reviewed studies have reported high rates of comorbidity between MDD and PTSD, more works are needed to have a better understanding of this relationship. For example, an interesting objective would be to examine the form in which both pathologies vary over the time after terrorism. In this line, some authors have recently documented the important role that depressive symptoms plays in the development and persistence of stress post-traumatic symptoms after different traumatic events [62]. Fourth, some of the studies in this revision included victims who had been bereaved [33,37]. Although not reported in these papers, differences in the prevalence of MDD may exist between victims who have been directly injured by a terrorist attack and those who have been bereaved. Moreover, bereaved people could develop other psychological problems, such as complicated grief syndrome. A number of studies support the differentiation between complicated grief and MDD [63,64], and some authors have shown that it is a usual reaction in bereaved people after terrorism [65]. A clear definition of victims in future works could provide us with a better understanding of the psychological consequences in people directly and strongly exposed to terrorism.

## Conclusions

The studies reviewed here, together with future research efforts in this field, should help to inform planned public mental health response that aims to mitigate the consequences of terrorist attacks by estimating the possible number of persons with MDD after such attacks, the potential course of the psychopathological burden, and the detection of populations at risk of developing these problems.

## List of abbreviations used

MDD: Major Depressive Disorder; DSM: Diagnostic and Statistical Manual of Mental Disorders; M-11: March 11, 2004 terrorist attacks in Madrid; S-11: September 11, 2001 terrorist attacks in New York; PTSD: Posttraumatic Stress Disorder.

## Competing interests

The authors declare that they have no competing interests.

## Authors' contributions

JMSN, PFB, II and ACV were responsible for the conception and design of the study. JMSN and PFB performed the databases search and the initial revision of abstracts. JMSN, PFB and II reviewed the chosen studies in depth. JMSN was responsible for writing the drafts of this paper. ACV and SG were responsible for revising the paper critically for important intellectual content. All authors read and approved the final manuscript.

## Pre-publication history

The pre-publication history for this paper can be accessed here:

http://www.biomedcentral.com/1471-244X/11/96/prepub
